# Nomogram for the prediction of postoperative hypoxemia in patients with acute aortic dissection

**DOI:** 10.1186/s12871-018-0612-7

**Published:** 2018-10-20

**Authors:** Huiqing Ge, Ye Jiang, Qijun Jin, Linjun Wan, Ximing Qian, Zhongheng Zhang

**Affiliations:** 1 Department of Respiratory Care, Sir Run Run Shaw Hospital, Zhejiang University School of Medicine, Hangzhou, China; 2 Department of Cardiovascular Surgery, Sir Run Run Shaw Hospital, Zhejiang University School of Medicine, Hangzhou, China; 3 Department of Emergency Medicine, Sir Run Run Shaw Hospital, Zhejiang University School of Medicine, No 3, East Qingchun Road, Hangzhou, 310016 Zhejiang Province China

**Keywords:** Acute aortic dissection, Hypoxemia, Nomogram, Intensive care unit, Length of stay

## Abstract

**Background:**

Postoperative hypoxemia is quite common in patients with acute aortic dissection (AAD) and is associated with poor clinical outcomes. However, there is no method to predict this potentially life-threatening complication. The study aimed to develop a regression model in patients with AAD to predict postoperative hypoxemia, and to validate it in an independent dataset.

**Methods:**

All patients diagnosed with AAD from December 2012 to December 2017 were retrospectively screened for potential eligibility. Preoperative and intraoperative variables were included for analysis. Logistic regression model was fit by using purposeful selection procedure. The original dataset was split into training and validating datasets by 4:1 ratio. Discrimination and calibration of the model was assessed in the validating dataset. A nomogram was drawn for clinical utility.

**Results:**

A total of 211 patients, involving 168 in non-hypoxemia and 43 in hypoxemia group, were included during the study period (incidence: 20.4%). Duration of mechanical ventilation (MV) was significantly longer in the hypoxemia than non-hypoxemia group (41(10.5140) vs. 12(3.75,70.25) hours; *p* = 0.002). There was no difference in the hospital mortality rate between the two groups. The purposeful selection procedure identified 8 variables including hematocrit (odds ratio [OR]: 0.89, 95% confidence interval [CI]: 0.80 to 0.98, *p* = 0.011), PaO_2_/FiO_2_ ratio (OR: 0.99, 95% CI: 0.99 to 1.00, *p* = 0.011), white blood cell count (OR: 1.21, 95% CI: 1.06 to 1.40, *p* = 0.008), body mass index (OR: 1.32, 95% CI: 1.15 to 1.54; *p* = 0.000), Stanford type (OR: 0.22, 95% CI: 0.06 to 0.66; *p* = 0.011), pH (OR: 0.0002, 95% CI: 2*10^− 8^ to 0.74; *p* = 0.048), cardiopulmonary bypass time (OR: 0.99, 95% CI: 0.98 to 1.00; *p* = 0.031) and age (OR: 1.03, 95% CI: 0.99 to 1.08; *p* = 0.128) to be included in the model. In an independent dataset, the area under curve (AUC) of the prediction model was 0.869 (95% CI: 0.802 to 0.936). The calibration was good by visual inspection.

**Conclusions:**

The study developed a model for the prediction of postoperative hypoxemia in patients undergoing operation for AAD. The model showed good discrimination and calibration in an independent dataset that was not used for model training.

## Background

Acute aortic dissection (AAD) represents a life-threatening condition that can be encountered in emergency and critical care setting [1]. Many factors can influence the clinical outcomes of these patients such as the comorbidities, complications, organ dysfunction and site of dissection. Surgical operation is usually needed to avert catastrophic complications of aortic dissection [2]. Postoperative hypoxemia has long been noted in substantial proportion of patients with AAD and has been found to be associated with poor clinical outcomes such as prolonged mechanical ventilation, increased length of stay (LOS) in the intensive care unit (ICU) and hospital [3–5]. Also, several studies have attempted to identify preoperative risk factor of hypoxemia [4, 5]. However, there is no report on training a model for early prediction of postoperative hypoxemia. Since early prediction of post-operative hypoxemia makes early intervention possible, it is of clinical utility to train and validate such a prediction model. The study aimed to develop a model for early prediction of postoperative hypoxemia. Discrimination and calibration of the model were validated in an independent dataset that was not used for model training. A nomogram was depicted for clinical use.

## Methods

### Study design and settings

The study was retrospective in design. All patients diagnosed as AAD from December 2012 to December 2017 were screened for potential eligibility. The patients were identified from the electronic healthcare record (EHR) of our hospital. Patients with initial suspected diagnosis of AAD as denoted by ICD9 code of 443.21 were identified. Exclusion criteria included one of the following items: 1) patients did not undergo surgery; 2) patients who were pregnant, or had neuromuscular disease, 3) confirmed complications such as heart failure, massive bleeding, pneumothorax, tracheal hemorrhage, atelectasis and pneumonia; and 3) patients had missing values on more than 50% variables. Data were extracted from EHR and deidentified before analysis. The study was approved by the ethics committee of Sir Run Run Shaw hospital (20180611–7). Informed consent was waived due to retrospective nature of the study.

### Variables included for analysis

Demographic variables including age, gender, body weight, height, body mass index (BMI) were obtained for the hospital admission with surgical repair of the aorta artery. Past histories of smoking, hypertension and diabetes mellitus were also included. The admission type included emergency and non-emergency admissions.

Preoperative laboratory tests were obtained within 24 h before surgery, which included albumin, hematocrit (HCT), pH, lactate, PaCO2, PaO2/FiO2 ratio (P/F), serum creatinine (Scr), total bilirubin, white blood cell count (WBC), C-reactive protein (CRP), troponin, creatine kinase (CK), creatine kinase isoenzymes (CKMB), lactate dehydrogenase (LDH), aspartate aminotransferase (AST). If there were two or more measures of these variables before operation, the one nearest to the operation was employed.

Intraoperative variables included aortic clamping time, cardiopulmonary bypass (CPB) time, duration of the operation, fluid input and output during operation, and the minimum body temperature.

Clinical outcomes were LOS in ICU and hospital, duration of postoperative mechanical ventilation, and hospital mortality.

Postoperative hypoxemia was defined as P/F < 200 for the first 2 days after operation.

### Statistical analysis

Continuous variables were expressed as mean and standard deviation for normally distributed data, and as median and interquartile range (IQR) for non-normal data. Categorical variables were expressed as number and percentage. Comparisons between hypoxemia and non-hypoxemia groups were performed using student t test or rank sum test as appropriate. Chi-square or Fisher’s exact test was employed for categorical variables [6, 7].

Postoperative hypoxemia was employed as response variable assuming a binomial distribution, and covariates were included in the model if their *p* values were less than 0.05 in univariate analysis [8]. Other variables such as age, CPB time and pH were entered due to clinical expertise. Variables with *p* > 0.2 in the multivariable model were excluded. The initial dataset was randomly split into the training and validating dataset by 4:1 ratio (there were 36 cases of hypoxemia in the training set). The training set was used to develop the model, and the validating set was used to validate the model. Model discrimination was represented by the area under receiver operating characteristic curves (AUC), with an AUC greater than 0.8 indicating a good discrimination [9]. Model calibration was visually assessed by plotting fitted logistic calibration curve and a smooth nonparametric fit using lowess and grouped proportions vs. mean predicted probability in group [10]. Other statistics were also reported with the R package *Regression Modeling Strategies* (*rms)*, such as Somers’D_{xy}, rank correlation between predicted probability and observed probability, Nagelkerke-Cox-Snell-Maddala-Magee R-squared index, the 0.9 quantile of same (E90), the Spiegelhalter Z-test for calibration accuracy, and its two-tailed *P*-value [11, 12].

A nomogram was drawn based on the fitted logistic regression model and each patient could be mapped onto the nomogram for the prediction of the occurrence of postoperative hypoxemia [13]. We drew the nomogram that converts each effect in the model to a 0 to 100 scale which is just proportional to the log odds. These points are added across predictors to derive the “Total Points,” which are converted to linear predictor and then to predicted probabilities [12]. The distribution of covariates in the model, and of the total regression score, are superimposed on the nomogram scales. Also, the values of a sample patient were superimposed. Binomial distribution with logit link function was employed for the model fit.

All statistical analyses were performed using R (version 3.4.3). Two-tailed *p* value less than 0.05 was considered as statistical significance.

## Results

### Patient inclusion

**Fig. 1:**
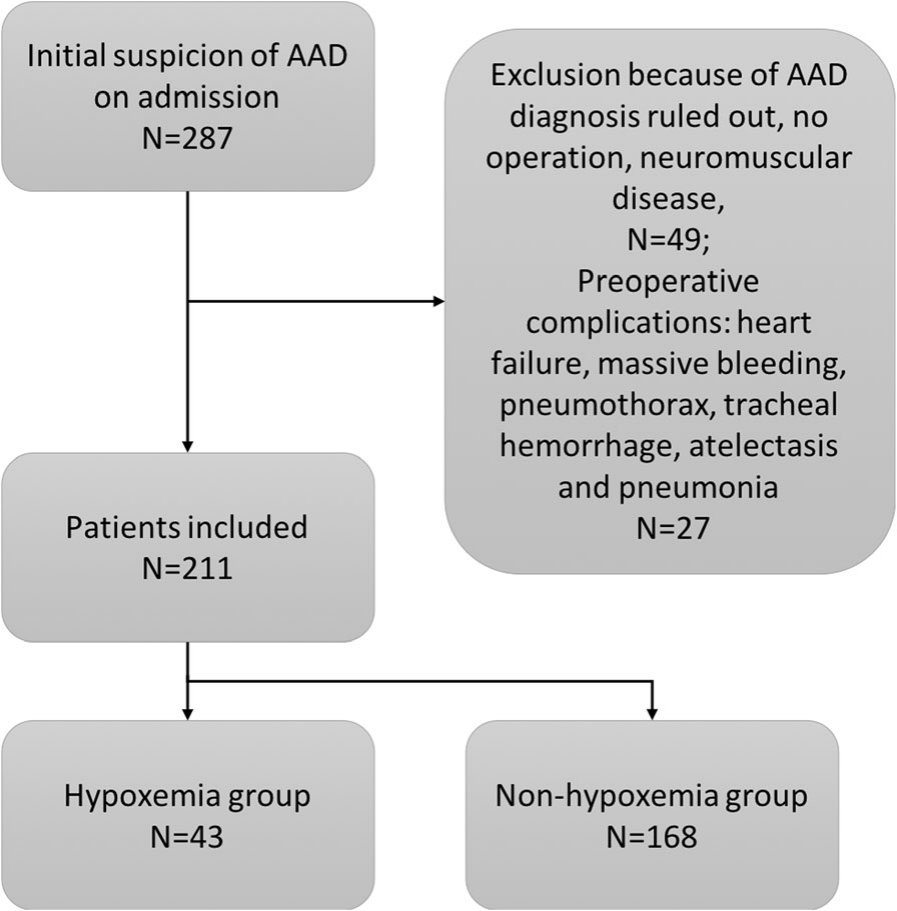
Flow chart of patient selection

A total of 287 patients with initial suspicion of AAD were initially identified from HER, and 49 were excluded because they did not undergo surgery during hospital stay, were ruled out for AAD, had neuromuscular disease. Furthermore, we excluded 27 patients with preoperative complications such as heart failure, massive bleeding, pneumothorax, atelectasis and pneumonia. As a result, a total of 211 patients were finally included for analysis (Fig. 1).

### Baseline characteristics

There were 168 patients in the non-hypoxemia group and 43 in the hypoxemia group, with an incidence rate of 20.4%. Patients in the hypoxemia group appears to be elder than non-hypoxemia group, but the statistical significance was not reached (Table 1). Patients with hypoxemia showed significantly greater BMI than those in non-hypoxemia group (26.82 ± 3.84 vs. 24.94 ± 3.86 kg/m2, *p* = 0.006). All patients (100%) with hypoxemia were admitted from emergency setting, versus 86% for the non-hypoxemia group (*p* = 0.005).

**Table 1:** Comparison between hypoxemia and non-hypoxemia groups

	Total (*n* = 211)	Non-hypoxemia (*n* = 168)	Hypoxemia (*n* = 43)	p
Demographics				
Age, median (IQR) (years)	50 (43,63)	50 (42,64)	52 (46.5,59)	0.654
Male, n (%)	151 (0.72)	116 (0.69)	35 (0.81)	0.158
Height, median (IQR) (cm)	170 (163,174)	170 (162.75,174)	169 (165,174)	0.647
Weight, median (IQR) (kg)	70 (62,80)	70 (60,80)	80 (65,89.5)	0.005
BMI, mean ± SD (kg/m2)	25.32 ± 3.92	24.94 ± 3.86	26.82 ± 3.84	0.006
Smoking history, n (%)	59 (28)	47 (28)	12 (28)	0.998
Hypertension, n (%)	134 (64)	102 (61)	32 (74)	0.137
Diabetes, n (%)	13 (6)	11 (7)	2 (5)	0.998
Stanford A, n (%)	133 (63)	97 (0.58)	36 (84)	0.003
Emergency admission, n (%)	188 (89)	145 (86)	43 (1)	0.005
Laboratory tests before operation				
Albumin, mean ± SD (mg/l)	36.6 ± 4.65	36.75 ± 4.76	36 ± 4.22	0.316
Hematocrit, mean ± SD (%)	36.09 ± 5.71	36.26 ± 5.4	35.41 ± 6.82	0.452
pH, median (IQR)	7.37 (7.34,7.4)	7.37 (7.34,7.4)	7.37 (7.32,7.38)	0.205
Lactate, median (IQR) (mmol/l)	2.1 (1.5,2.9)	2.05 (1.48,2.8)	2.30 (1.9,3.1)	0.082
PaCO2, median (IQR) (mmHg)	41 (37.55,45.55)	41 (37.88,45.23)	40.9 (36.5,47.7)	0.916
P/F ratio, median (IQR)	235 (176,355)	249 (183.75,367.25)	174 (148.5237)	< 0.001
Scr, median (IQR) (mmol/l)	81 (64,113)	76.5 (61,104)	103 (77.5145)	< 0.001
Total bilirubin, median (IQR) (mmol/l)	13.7 (10,22.25)	13.2 (9.57,18.9)	16.7 (11.9,25)	0.016
WBC, median (IQR) (^109/l)	9.5 (7.4,12.6)	8.9 (6.8,11.65)	12.9 (9.3,15.5)	< 0.001
CRP, median (IQR) (mg/l)	28.3 (5.75,68.75)	23.4 (4.57,60.38)	58.9 (10.6118.3)	0.003
Troponin, median (IQR) (ng/ml)	0.01 (0.01,0.01)	0.01 (0.01,0.01)	0.01 (0.01,0.12)	0.028
CK, median (IQR) (U/l)	78 (51,150.5)	72 (48.75,136.25)	109 (62,228)	0.023
CKMB, median (IQR) (U/l)	10 (8,16)	10 (8,16)	10 (6.5,14.5)	0.249
LDH, median (IQR) (U/l)	218 (171,283)	208.5 (165,274.25)	253 (196.5312.5)	0.013
AST, median (IQR) (U/l)	22 (16.5,34.5)	21 (15,31.25)	31 (18.5,44.5)	0.007
Intraoperative variables				
Aortic clamping time, mean ± SD (min)	127.91 ± 40.82	128.84 ± 40.7	124.26 ± 41.59	0.519
CBP time, median (IQR) (min)	178 (140,211)	180 (146.75,210)	169 (132.5221)	0.749
Duration of operation, median (IQR) min	295 (120,390)	285 (90,390)	300 (242.5420)	0.072
Minimum temperature, median (IQR) (°C)	36.2 (35.6,36.55)	36.2 (35.4,36.6)	36.3 (36,36.5)	0.250
Input, median (IQR) (ml)	6250 (5150,7587.5)	6090 (5150,7332.5)	6380 (4775,8170)	0.651
Output, median (IQR) (ml)	4850 (3600,5800)	4900 (3575,6000)	4500 (3675,5600)	0.358
Clinical outcomes				
LOS in ICU, median (IQR) (days)	7 (4,12)	7 (4,11)	10 (5.5,14)	0.079
LOS in hospital, median (IQR) (days)	19 (13,24.5)	19 (13,25)	18 (12.5,24)	0.775
Duration of MV, median (IQR) (hours)	15 (5,82)	12 (3.75,70.25)	41 (10.5140)	0.002
Mortality, n (%)	7 (3)	6 (4)	1 (2)	0.997

Note: continuous variables were expressed as mean and standard deviation for normal data, and as median and interquartile range for non-normal data. Categorical variables were expressed as number and percentage. Comparisons between hypoxemia and non-hypoxemia groups were performed using student t test or rank sum test as appropriate. Chi-square or Fisher’s exact test was employed for categorical variables

*Abbreviations*: *No.* number, *Prop.* proportion, *ICU* intensive care unit, *LOS* length of stay, *WBC* white blood cell count, *CRP* c-reactive protein, *Scr* serum creatinine, *BMI* body mass index, *CK* creatine kinase, *CKMB* creatine kinase isoenzymes, *LDH* lactate dehydrogenase, *AST* aspartate aminotransferase, *MV* mechanical ventilation, *CPB* cardiopulmonary bypass, *IQR* interquartile range, *SD* standard deviation

### Preoperative laboratory test

For preoperative laboratory tests, hypoxemia patients showed significantly lower P/F ratio than non-hypoxemia patients (median, interquartile range [IQR]: 174(148.5237) vs. 249(183.75,367.25); *p* < 0.001). Other laboratory tests such as serum creatinine (103(77.5145) vs. 76.5(61,104) mmol/l; *p* < 0.001), total bilirubin (16.7(11.9,25) vs. 13.2(9.57,18.9) mmol/l; *p* = 0.016), WBC (12.9(9.3,15.5) vs. 8.9(6.8,11.65) ^10^9^/l; *p* < 0.001), CRP (58.9(10.6118.3) vs. 23.4(4.57,60.38) mg/l; *p* = 0.003) and CK (109(62,228) vs. 72(48.75,136.25) U/l; *p* = 0.023) were significantly higher in the hypoxemia group than that in the non-hypoxemia group.

Intraoperative variables were not significantly different between hypoxemia and non-hypoxemia groups.

### Clinical outcomes

The LOS in ICU (10(5.5,14) vs. 7(4,11) days; *p* = 0.079) and hospital (18(12.5,24) vs. 19(13,25) days; *p* = 0.775) were not significantly different between the two groups. Duration of MV was significantly longer in the hypoxemia than non-hypoxemia group (41(10.5140) vs. 12(3.75,70.25) hours; *p* = 0.002). There was no difference in the hospital mortality rate between the two groups (Table 1).

### Model training

Logistic regression model was fit with the training set. The purposeful selection procedure included 8 variables as determined by clinical importance and statistical significance (Table 2). The preoperative HCT (odds ratio [OR]: 0.89, 95% confidence interval [CI]: 0.80 to 0.98, *p* = 0.011), P/F ratio (OR: 0.99, 95% CI: 0.99 to 1.00, *p* = 0.011), WBC (OR: 1.21, 95% CI: 1.06 to 1.40, *p* = 0.008), BMI (OR: 1.32, 95% CI: 1.15 to 1.54; *p* < 0.001), Stanford type (OR: 0.22, 95% CI: 0.06 to 0.66; *p* = 0.011), pH (OR: 0.0002, 95% CI: 2*10^− 8^ to 0.74; *p* = 0.048), and CPB time (OR: 0.99, 95% CI: 0.98 to 1.00; *p* = 0.031) were significantly contributing to the prediction accuracy of the model. Age was not significantly associated with the outcome, but it remained in the model by clinical judgement.

**Table 2:** Logistic regression model for the prediction of postoperative hypoxemia

Variables	Odds ratio	Lower limit of 95% CI	Upper limit of 95% CI	*P* value
BMI	1.32	1.15	1.54	< 0.001
PF	0.99	0.99	1.00	0.011
Stanford (A as reference)	0.22	0.06	0.66	0.011
WBC	1.21	1.06	1.40	0.008
Age	1.03	0.99	1.08	0.128
HCT	0.89	0.80	0.98	0.016
CBP time	0.99	0.98	1.00	0.031
pH	0.0002	2*10^−8^	0.74	0.048

Note: The logistic regression model was selected by using stepwise forward selection procedure, AIC was used to decide the inclusion of a variable. The odds ratio was reported for each one unit increase for each variable

*Abbreviations*: *CI* confidence interval, *WBC* white blood cell count, *PF* PaO2/FiO2, *BMI* body mass index, *CBP* cardiopulmonary bypass, *HCT* hematocrit

### Model validation in a separate dataset

**Fig. 2:**
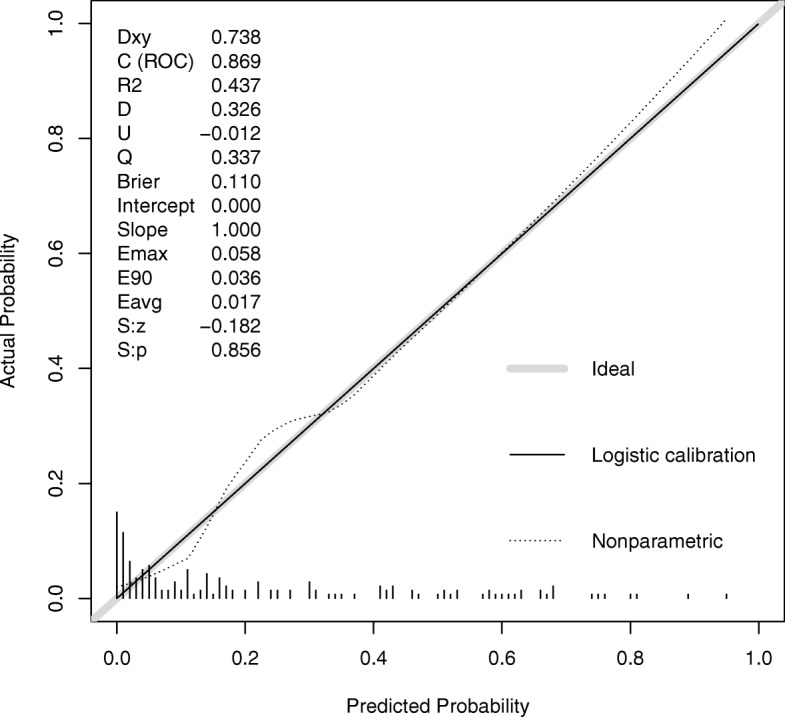
Model calibration and discrimination. The predicted probability by the logistic regression model conforms well to the observed probability well. In the plot, the idea line is consistent with the Logistic calibration line. The discrimination was measured by AUC of 0.869 (95% CI: 0.802 to 0.936)

**Fig. 3:**
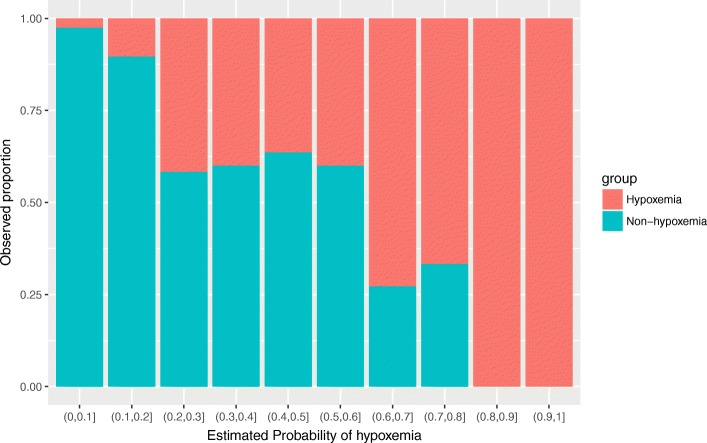
Bar chart showing the agreement between predicted probability of hypoxemia and observed proportion

In the validation dataset, the fitted model showed a good discrimination in distinguishing hypoxemia from non-hypoxemia patients (area under curve [AUC] = 0.869, 95% CI: 0.802 to 0.936). Model calibration was shown in Fig. 2. The non-parametric curve fits well to the ideal line, indicating the observed probability was in line with the predicted probability. However, the model may not predict well for patients with lower risk of postoperative hypoxemia. Also, the Fig. 3 shows that the predicted probability of hypoxemia is in agreement with the observed proportion.

### Nomogram for predicting postoperative hypoxemia

**Fig. 4:**
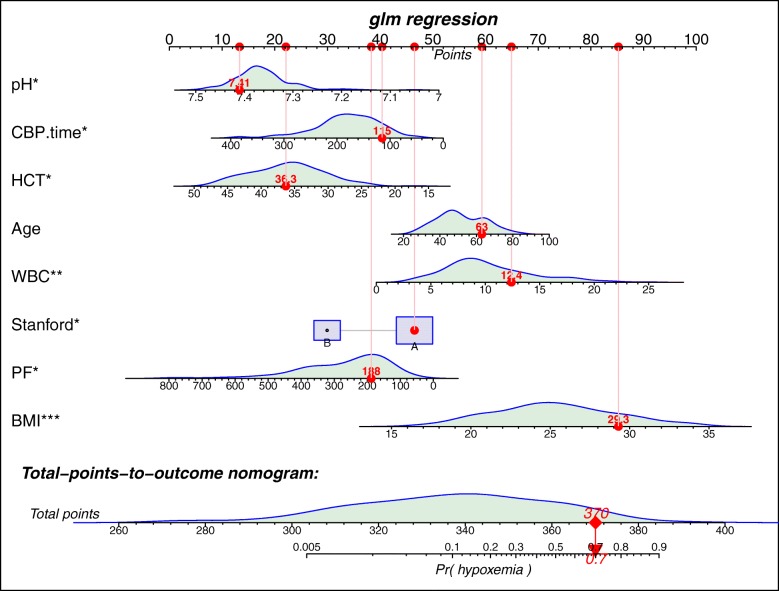
Nomogram for the prediction of postoperative hypoxemia. The logistic regression model were described as a series of straight lines with a common linear scale in the nomogram, with the scale factors of the individual lines given by the coefficients (beta) of the covariates in the model.The distribution of each variable is superimposed on each scale. A representative patient was shown to illustrate how to use the nomogram. Given values of the eight predictors, the patient can be mapped onto the nomogram. Note there is a red dot at each scale, representing the value of each of the 8 predictors for the patient. The total point of the patient was 370, corresponding to a probability of 0.7 for developing postoperative hypoxemia

Nomogram for the prediction of postoperative hypoxemia is shown in Fig. 4. The distribution of each variable was shown above each line. A representative patient was shown to illustrate how to use the nomogram. Given values of the 8 predictors, the patient can be mapped to the nomogram. Note there is a red dot in each line, representing the value of each of the 8 predictors for the patient. Regression coefficient of each predictor was scaled to points within the range of 0 to 100, and the relative importance (weight) of each predictor can be reflected by its points. Points was translated into probability by logit transformation.

## Discussion

The study included patients with AAD who had undergone operation for the repair of the dissection. Risk factors for postoperative hypoxemia were identified via purposeful selection procedure. These factors included age, lactate, preoperative P/F ratio, WBC, BMI and CRP. Internal validation was performed for the model, which showed good discrimination and calibration. A nomogram was established for clinical utility.

The incidence of hypoxemia was lower than those reported in the literature (20% vs. 30%) [4, 14]. Different definitions of postoperative hypoxemia may explain its different incidences. For example, Wang’s study defined hypoxemia as P/F ratio less than 200 at 24 h after operation and they reported an incidence of 49.5% [5]. Several studies have been conducted to explore risk factors for postoperative hypoxemia. Consistent with our study, the study by Liu and colleagues also showed the preoperative P/F ratio and WBC were independently associated with postoperative hypoxemia [4]. It is not surprising that preoperative P/F ratio is independently associated with postoperative hypoxemia. Furthermore, WBC is a biomarker reflecting systemic inflammatory response, and higher responses may contribute to the respiratory dysfunction. There is empirical evidence that inflammatory response is associated with hypoxemia in patients with aortic dissection [15]. Furthermore, we also found CRP was associated with hypoxemia. CRP is a well-known biomarker of inflammatory response, which is more specific and sensitive than WBC [16, 17]. However, the effect of CRP disappeared after adjusting for covariates. Liu’s study also included time from symptom onset and deep hypothermic circulatory arrest time in their regression model. None of the intraoperative variables were associated with hypoxemia in univariate analysis, but the CPB time was associated with hypoxemia in multivariable regression model. BMI was identified as an independent predictor of postoperative hypoxemia in the study, consistent with the study by Sheng and colleagues [14]. Actually, the association of obesity and hypoxemia is not limited to aortic surgery [18]. In a large cohort of noncardiac surgery, Kendale SM and colleagues found that the odds of experiencing hypoxemia increased significantly with increasing categories of BMI [19]. Similar results were documented in other studies [20, 21]. BMI is an important determinant of respiratory function and studies show morbidly obese patients have a typical restrictive pattern with a reduction of forced vital capacity (FVC), forced residual capacity (FRC) and total lung capacity (TLC) with a decreased expiratory reserve volume (ERV) [22–24]. Sex has been found to be related to postoperative hypoxemia in AAD (women were more likely to have hypoxemia than man), which was not replicated in our study. Most probably, the associated was confounded by other factors and the authors failed to adjust for these potential confounding factors [25].

An interesting finding in our study was that serum lactate was associated with postoperative hypoxemia in patients with AAD. Hyperlactatemia is an indicator of tissue ischemia [26]. In cardiac surgery patients, there is evidence that hyperlactatemia is associated with a compromised respiratory function and prolonged mechanical ventilation. Also, hyperlactatemia can explain organ dysfunction in our study [27–29]. However, the effect of lactate disappeared in multivariable model, indicating that the effect could be explained by preoperative P/F ratio, as lactate was a biomarker of hypoxemia. Similarly, preoperative P/F ratio can explain the elevations of biomarkers of acute organ injury such as Scr, bilirubin and ALT. That was why the significant associations in univariate analysis disappeared in the multivariable regression model.

The potential utility of our prediction model is that interventions such as anti-inflammatory agents can be given to patients with high risk of postoperative hypoxemia. Furthermore, the model can be employed to design clinical trials to identify high risk patients who will benefit the most from treatment [30].

There were some limitations in the present study that must be acknowledged. First, the study was retrospective single center study. Although the prediction model was validated in a separate dataset that was not used for model training, its external validity was still unknown. In different cardiac centers, the healthcare process can be quite different and the predictive accuracy of a newly developed model needs to be tested [31, 32]. This is also our future work to perform a multicenter study to examine the external validity of the model. Second, postoperative hypoxemia reported in the study was defined by P/F ratio less than 200 for consecutive 2 days. While this definition was simple to perform, it suffers from case-mix. Some patients may have intensive respiratory support, and their P/F ratio is not comparable to those with spontaneous breathing. Thus, the definition of postoperative hypoxemia needs to be further explored. However, in order to make our results comparable to that in the literature [4], we opt to use this simple definition. Third, the retrospective design of the study suffers from its inherent limitation of selection bias. Some patients were excluded because they did not perform operation in our hospital. They might go to other hospital for further evaluation. Thus, the included patients may not well represent the whole target population of AAD, but they represent those who are willing to undergo operation in our hospital. Finally, the sample size of the study was small that only 43 patients had the event of interest. As a rule of thumb, 10–20 events per variable should be used [33]. However, the primary purpose of this rule is to prevent overfitting [34]. We addressed the problem of overfitting by validating the model in a dataset that was not used for model training (e.g. an overfitting model performs poorly in the validation dataset). The results showed that the developed model can predict accurately in the validation dataset with an AUC of 0.869. Further studies by employing multicenter data are mandatory to endure generalizability of the model.

## Conclusion

In conclusion, the study developed a model for the prediction of postoperative hypoxemia in patients undergoing operation for AAD. Eight variables of age, BMI, WBC, HCT, CPB time, pH, Stanford type and P/F ratio were included in the model. The model showed good discrimination and calibration in an independent dataset that was not used for model training.

## Abbreviations

AST: Aspartate aminotransferase
BMI: Body mass index
CK: Creatine kinase
CKMB: Creatine kinase isoenzymes
CPB: Cardiopulmonary bypass
CRP: C-reactive protein
ICU: Intensive care unit
LDH: Lactate dehydrogenase
LOS: Length of stay
MV: Mechanical ventilation
Scr: Serum creatinine
WBC: White blood cell count
